# A PDE4 shortform degrader: a first in isoform‐specific PDE4 inhibition

**DOI:** 10.1111/febs.70059

**Published:** 2025-03-07

**Authors:** Donald H. Maurice

**Affiliations:** ^1^ Department of Biomedical and Molecular Sciences Queen's University Kingston Canada

**Keywords:** cAMP‐signaling, PDE4, phosphodiesterases, PROTAC

## Abstract

Although phosphodiesterase 4 (PDE4) inhibitors have reached the clinic, their lack of selectivity for PDE4 enzyme isoforms leads to documented side effects. Building in enzyme selectivity has proved difficult because all PDE4 enzymes share highly conserved catalytic domains. The report by Sin *et al.* describes a novel approach in which a potent PDE4 proteolysis targeting chimera (PROTAC) selectively promotes the degradation of a small subset of PDE4 isoforms (*i.e.,* “short forms”) and impacts inflammatory events regulated by these enzymes. This approach offers unparalleled selectivity, potency, and could represent the dawn of a new pharmacology for selective regulation of cyclic AMP (cAMP) signaling.

## Graphical abstract

Clinically used phosphodiesterase 4 (PDE4) inhibitors lack selectivity for PDE4 enzyme isoforms, which leads to documented side effects. Building in enzyme selectivity has proved difficult because all PDE4 enzymes share highly conserved catalytic domains. The report by Sin *et al.* describes a novel approach in which a potent PDE4 proteolysis targeting chimera (PROTAC) selectively promotes the degradation of a small subset of PDE4 isoforms (i.e., “short forms”) and impacts inflammatory events regulated by these enzymes. This approach offers unparalleled selectivity, potency, and could represent the dawn of a new pharmacology for selective regulation of cyclic AMP (cAMP) signaling.

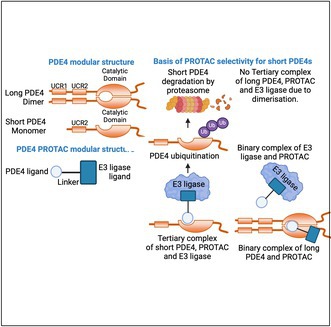

**Abbreviations**
cAMP: cyclic adenosine monophosphate
PDE4: phosphodiesterase 4
POI: protein of interest
PROTAC: proteolysis targeting chimera

Cyclic AMP (cAMP) is an archetypal second messenger that controls cellular homeostasis in most cells. Since aberrant cell signaling caused by the paucity of this freely diffusible chemical agent often results in disease, numerous strategies aimed at elevating cAMP levels in cells have been developed. For example, inhibition of cAMP‐specific phosphodiesterase 4 (PDE4) activity using small molecules that block access of cAMP to its binding site raises cAMP concentrations in several cell types, provides clinical utility in a range of inflammatory diseases, and shows great potential in preclinical models and clinical trials for respiratory diseases, skin conditions, and rheumatoid arthritis [1]. Although PDE4 inhibition shows promise, one important drawback of inhibiting PDE4 using small molecules targeting the active site is that all 25 PDE4 isoforms encoded by the four *PDE4* genes (*i.e*., *PDE4A, B, C* and *D*) share a high level of conserved sequence and structure in this region [2]. Indeed, this situation makes most PDE4 inhibitors in use “pan”‐PDE4 inhibitors and results in off‐target effects that limit their therapeutic window and clinical utility. While rapidly evolving *in silico* docking techniques have taken advantage of small differences in the active site residues between PDE4s to design more selective active site blockers, no agent yet developed has absolute specificity for any individual *PDE4* gene‐encoded enzyme or variant [3, 4]. Similarly, while allosteric compounds that dock to sites formed when regulatory and catalytic regions interact have allowed the development of “longform” selective agents, again, these agents show little selectivity between PDE4 family enzymes.

A novel pharmacological approach using targeted protein degradation is revolutionizing accessibility to previously recalcitrant drug targets [5]. In brief, a proteolysis targeting chimera (PROTAC) can drive the natural degradation of a protein via the ubiquitin proteasome system. Individual PROTACs utilize a “warhead,” which targets a protein of interest (POI) that is covalently linked to a carbon chain linker. To the other end of the linker is conjugated a moiety that recruits an E3 ubiquitin ligase to rapidly induce the degradation of the POI via the 26S proteasome (Fig. 1A). Targeted degradation in this manner has many advantages over occupancy‐based inhibition: (a) concomitantly reducing the enzymatic and nonenzymatic functions of the POI, (b) lack of “rescue” via *de novo* transcription/translation of target POI, (c) sequential degradation over time resulting in much lower doses being required, and (d) the ability to “build‐in” selectivity for single isoforms of similar proteins via the adaptability of the three component parts of the PROTAC (*i.e*., warhead, linker, and E3 ligase glue). The benefits of this PROTAC‐based approach have hastened the development of clinically ready candidates, mostly for oncology, which are now in late‐stage trials [6]. The aptness of targeted degradation for the development of PDE inhibitors is obvious, and one earlier attempt produced a selective degrader of PDE6δ, a promiscuous prenyl‐binding protein, originally identified as a PDE6 subunit, that was effective against cancers driven by mutated KRAS [7]. Similarly, a separate PROTAC was shown to promote limited (50%) degradation of a PDE4A target, although this earlier report did not discuss its PDE4A selectivity [8].

**Fig. 1:**
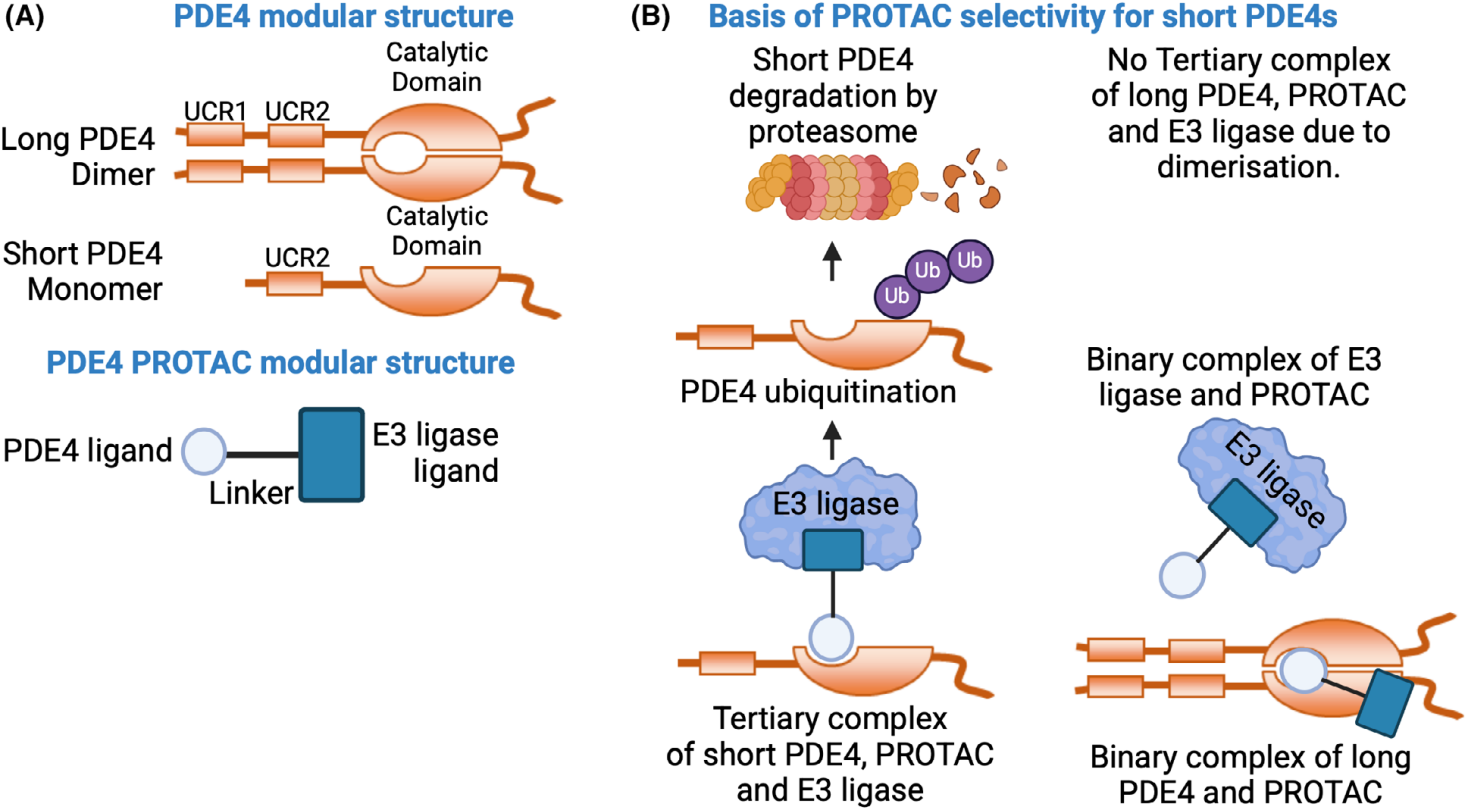
The PROTAC modular structure and basis of PROTAC selectivity for short PDE4s. (A) Upper panel: Modular structure of PDE4s outlining the difference between long PDE4 isoforms that contain Upstream conserved region 1 (UCR1) and Upstream conserved region 2 (UCR2) and short PDE4 isoforms that lack UCR1. This structural difference inhibits the dimerization of PDE4 shortforms. Lower panel: A schematic that illustrates the functional domains of a PDE4 PROTAC. (B) A schematic showing the proposed mechanism of selectivity of the PROTAC for short form PDE4s. Due to the dimerization of longforms, a tertiary complex cannot form to direct the ubiquitination of this PDE4 class. PDE4 shortforms can spatially accommodate the E3 ligase, leading to ubiquitination and subsequent degradation of these isoforms by the proteasome.

The data described by Sin *et al*. [9] represent a marked advance from this pioneering work. Thus, the PROTAC (KTX207) had high selectivity for PDE4 shortforms over longform variants and was completely specific for PDE4D shortforms when used at low concentrations (10 pM) (Fig. 1B). This level of specificity is unprecedented in the field of PDE4 inhibitors. Indeed, clever modeling of the trimeric complex (PDE4, PROTAC, and E3 ligase) and alterations to the linker region which allowed E3 ligase recruitment to monomeric PDE4s (*i.e*., shortforms), but not dimeric forms (*i.e*., longforms) (Fig. 1B), resulted in a compound that degrades only a small subset (6 shortforms) of the 25 *PDE4* gene products. Other features that make KTX207 uniquely suitable for possible clinical use are its high potency (IC50: 10 pM), allowing low dosing and long‐lasting effects. Indeed, Sin and colleagues [9] showed that KTX207 caused long‐lasting and profound anti‐inflammatory effects which were directly related to the known PDE4 shortform upregulation that occurs in activated T cells [10].

Based on the findings of Sin *et al*. [9], one can predict that KTX207 would likely have utility in promoting long‐lasting cAMP signaling in all cells in which the upregulated expression of PDE4 short forms is functionally antagonistic. For example, since my laboratory reported that PDE4D2 upregulation antagonized the antiproliferative and antimigratory effects of cAMP‐elevating agents in the “activated” vascular smooth muscle cells (VSMCs), which promote restenosis of arteries post balloon angioplasty, I predict that KTX207 could find utility in reversing this desensitization and limiting restenosis. Further, since the nonactivated VSMCs not involved in restenosis do not express PDE4D short forms, I further predict that KTX207 would selectively limit restenosis and be without impact on blood vessel tone [11, 12].

A key concept to consider when using PROTACs to unhook PDE4 inhibition from side effects is cAMP compartmentation [13]. As agonists for many receptor types increase adenylyl cyclase synthesis of cAMP, the resulting shape and duration of the cAMP response are tailored by PDEs for intracellular selective responses. It is now established that targeted subcellular specific localization of individual PDE4 isoforms is crucial in shaping the three‐dimensional “cAMP cloud” that selectively activates the four known cAMP effector proteins [3, 13]. Indeed, if cAMP were allowed to flood the cell, every cAMP effector in all subcellular compartments would be activated simultaneously, obviating the signal deconvolution required for distinct localized signaling to occur. Hence, based on the results of Sin *et al*., it would seem reasonable to propose that isoform/sub‐family selective PROTACs aimed at specific PDE4 longform could greatly reduce “off‐compartment” side effects. For these PROTACs, warheads designed based on the longform allosteric modulators discussed above might represent reasonable candidates.

## Conclusion

Although progress may be hampered by the disadvantages of PROTAC development, which include lack of bioavailability, lack of penetrance to certain tissues/organs (e.g., brain), off‐target effects driven by sequestration of single E3 ligases, and lack of specific E3 expression in diseased tissue that is targeted [14], I propose that the targeted degradation route, so elegantly reported by Sin and colleagues, seems highly attractive for the design of “next generation” PDE4 inhibitors and that PDE4 enzymes may soon be found on the expanding list of targets for degraders that are reaching clinical trials.

## Conflict of interest

The authors declare no conflict of interest.

## References

[1] Crocetti L , Floresta G , Cilibrizzi A & Giovannoni MP (2022) An overview of PDE4 inhibitors in clinical trials: 2010 to early 2022. Molecules 27, 4964. 35956914 10.3390/molecules27154964 PMC9370432

[2] Paes D , Schepers M , Rombaut B , Van Den Hove D , Vanmierlo T & Prickaerts J (2021) The molecular biology of phosphodiesterase 4 enzymes as pharmacological targets: an interplay of isoforms, conformational states and inhibitors. Pharmacol Rev 73, 1016–1049. 34233947 10.1124/pharmrev.120.000273

[3] Maurice DH , Ke H , Ahmad F , Wang Y , Chung J & Manganiello VC (2014) Advances in targeting cyclic nucleotide phosphodiesterases. Nat Rev Drug Discov 13, 290–314. 24687066 10.1038/nrd4228 PMC4155750

[4] Baillie GS , Tejeda GS & Kelly MP (2019) Therapeutic targeting of 3′,5′‐cyclic nucleotide phosphodiesterases: inhibition and beyond. Nat Rev Drug Discov 18, 770–796. 31388135 10.1038/s41573-019-0033-4 PMC6773486

[5] Bekes M , Langley DR & Crews CM (2022) PROTAC targeted protein degraders: the past is prologue. Nat Rev Drug Discov 21, 181–200. 35042991 10.1038/s41573-021-00371-6 PMC8765495

[6] Wang X , Qin ZL , Li N , Jia MQ , Liu QG , Bai YR , Song J , Yuan S & Zhang SY (2024) Annual review of PROTAC degraders as anticancer agents in 2022. Eur J Med Chem 267, 116166. 38281455 10.1016/j.ejmech.2024.116166

[7] Cheng J , Li Y , Wang X , Dong G & Sheng C (2020) Discovery of novel PDEdelta degraders for the treatment of KRAS mutant colorectal cancer. J Med Chem 63, 7892–7905. 32603594 10.1021/acs.jmedchem.0c00929

[8] Ohoka N , Okuhira K , Ito M , Nagai K , Shibata N , Hattori T , Ujikawa O , Shimokawa K , Sano O , Koyama R *et al*. (2017) In vivo knockdown of pathogenic proteins via specific and nongenetic inhibitor of apoptosis protein (IAP)‐dependent protein erasers (SNIPERs). J Biol Chem 292, 4556–4570. 28154167 10.1074/jbc.M116.768853 PMC5377772

[9] Sin YY , Giblin A , Judina A , Rujirachaivej P , Corral LG , Glennon E , Tai ZX , Feng T , Torres E , Zorn A *et al*. (2025) Targeted protein degradation of PDE4 shortforms by a novel proteolysis targeting chimera. FEBS J 292, 3360–3377. 10.1111/febs.17359 PMC12220845 39673076

[10] Blauvelt A , Langley RG , Gordon KB , Silverberg JI , Eyerich K , Sommer MOA , Felding J & Warren RB (2023) Next generation PDE4 inhibitors that selectively target PDE4B/D subtypes: a narrative review. Dermatol Ther (Heidelb) 13, 3031–3042. 37924462 10.1007/s13555-023-01054-3 PMC10689637

[11] Tilley DG & Maurice DH (2002) Vascular smooth muscle cell phosphodiesterase (PDE) 3 and PDE4 activities and levels are regulated by cyclic AMP in vivo. Mol Pharmacol 62, 497–506. 12181425 10.1124/mol.62.3.497

[12] Tilley DG & Maurice DH (2005) Vascular smooth muscle cell phenotype‐dependent phosphodiesterase 4D short form expression: role of differential histone acetylation on cAMP‐regulated function. Mol Pharmacol 68, 596–605. 15937214 10.1124/mol.105.014126

[13] Baillie GS (2009) Compartmentalized signalling: spatial regulation of cAMP by the action of compartmentalized phosphodiesterases. FEBS J 276, 1790–1799. 19243430 10.1111/j.1742-4658.2009.06926.x

[14] Berkley K , Zalejski J , Sharma N & Sharma A (2025) Journey of PROTAC: from bench to clinical trial and beyond. Biochemistry 64, 563–580. 39791901 10.1021/acs.biochem.4c00577

